# Racial/Ethnic Differences in COVID-19 Vaccine Hesitancy Among Health Care Workers in 2 Large Academic Hospitals

**DOI:** 10.1001/jamanetworkopen.2021.21931

**Published:** 2021-08-30

**Authors:** Florence M. Momplaisir, Barbara J. Kuter, Fatemeh Ghadimi, Safa Browne, Hervette Nkwihoreze, Kristen A. Feemster, Ian Frank, Walter Faig, Angela K. Shen, Paul A. Offit, Judith Green-McKenzie

**Affiliations:** 1 Division of Infectious Diseases, Department of Medicine Perelman School of Medicine, University of Pennsylvania, Philadelphia; 2 Leonard Davis Institute of Health Economics, University of Pennsylvania, Philadelphia; 3 Vaccine Education Center, Children’s Hospital of Philadelphia, Philadelphia, Pennsylvania; 4 Division of Infectious Diseases, Department of Pediatrics, Perelman School of Medicine, University of Pennsylvania, Philadelphia; 5 Children's Hospital of Philadelphia Research Institute, Philadelphia, Pennsylvania; 6 Division of Occupational and Environmental Medicine, Department of Emergency Medicine, Perelman School of Medicine, University of Pennsylvania, Philadelphia

## Abstract

**Importance:**

Significant differences in hesitancy to receive COVID-19 vaccination by race/ethnicity have been observed in several settings. Racial/ethnic differences in COVID-19 vaccine hesitancy among health care workers (HCWs), who face occupational and community exposure to COVID-19, have not been well described.

**Objective:**

To assess hesitancy to COVID-19 vaccination among HCWs across different racial/ethnic groups and assess factors associated with vaccine hesitancy.

**Design, Setting, and Participants:**

This survey study was conducted among HCWs from 2 large academic hospitals (ie, a children’s hospital and an adult hospital) over a 3-week period in November and December 2020. Eligible participants were HCWs with and without direct patient contact. A 3-step hierarchical multivariable logistic regression was used to evaluate associations between race/ethnicity and vaccine hesitancy controlling for demographic characteristics, employment characteristics, COVID-19 exposure risk, and being up to date with routine vaccinations. Data were analyzed from February through March 2021.

**Main Outcomes and Measures:**

Vaccine hesitancy, defined as not planning on, being unsure about, or planning to delay vaccination, served as the outcome.

**Results:**

Among 34 865 HCWs eligible for this study, 12 034 individuals (34.5%) completed the survey and 10 871 individuals (32.2%) completed the survey and reported their race/ethnicity. Among 10 866 of these HCWs with data on sex, 8362 individuals (76.9%) were women, and among 10 833 HCWs with age data, 5923 individuals (54.5%) were younger than age 40 years. (Percentages for demographic and clinical characteristics are among the number of respondents for each type of question.) There were 8388 White individuals (77.2%), 882 Black individuals (8.1%), 845 Asian individuals (7.8%), and 449 individuals with other or mixed race/ethnicity (4.1%), and there were 307 Hispanic or Latino individuals (2.8%). Vaccine hesitancy was highest among Black HCWs (732 individuals [83.0%]) and Hispanic or Latino HCWs (195 individuals [63.5%]) (*P* < .001). Among 5440 HCWs with vaccine hesitancy, reasons given for hesitancy included concerns about side effects (4737 individuals [87.1%]), newness of the vaccine (4306 individuals [79.2%]), and lack of vaccine knowledge (4091 individuals [75.2%]). The adjusted odds ratio (aOR) for vaccine hesitancy was 4.98 (95% CI, 4.11-6.03) among Black HCWs, 2.10 (95% CI, 1.63-2.70) among Hispanic or Latino HCWs, 1.48 (95% CI, 1.21-1.82) among HCWs with other or mixed race/ethnicity, and 1.47 (95% CI, 1.26-1.71) among Asian HCWs compared with White HCWs (*P* < .001). The aOR was decreased among Black HCWs when adjusting for employment characteristics and COVID-19 exposure risk (aOR, 4.87; 95% CI, 3.96-6.00; *P* < .001) and being up to date with prior vaccines (aOR, 4.48; 95% CI, 3.62-5.53; *P* < .001) but not among HCWs with other racial/ethnic backgrounds.

**Conclusions and Relevance:**

This study found that vaccine hesitancy before the authorization of the COVID-19 vaccine was increased among Black, Hispanic or Latino, and Asian HCWs compared with White HCWs. These findings suggest that interventions focused on addressing vaccine hesitancy among HCWs are needed.

## Key Points

**Question:**

What are the differences in COVID-19 vaccine hesitancy by race/ethnicity among health care workers (HCWs)?

**Findings:**

This survey study of 10 871 HCWs from 2 academic hospitals found that, compared with White HCWs, vaccine hesitancy was increased nearly 5-fold among Black HCWs, 2-fold among Hispanic or Latino HCWs, and by nearly 50% among Asian HCWs and HCWs who were members of other racial/ethnic groups.

**Meaning:**

These findings suggest that interventions focused on addressing vaccine hesitancy among HCWs are needed, particularly for Black and Hispanic or Latino HCWs, among whom hesitancy is highest.

## Teaser

This survey study investigates the differences in COVID-19 vaccine hesitancy by race/ethnicity among health care workers at 2 large academic hospitals.

## Introduction

It has been more than a year since the first case of COVID-19 was reported in the US, and as of June 2021, more than 30 million diagnoses of COVID-19 and 590 000 deaths have been attributed to this outbreak.^1,2^ Although the pandemic has impacted everyone, COVID-19 disease burden disproportionately falls on members of racial/ethnic minority groups. Black and Hispanic or Latino individuals are nearly 3-fold more likely to be hospitalized owing to COVID-19 and approximately 2-fold more likely to die from the disease compared with White non-Hispanic individuals.^3^ With 3 authorized SARS-CoV-2 vaccines available for emergency use in the U.S,^4,5,6^ it is important to increase access to COVID-19 vaccines and address COVID-19 vaccine hesitancy in communities where it is still high.^7,8^

The vaccine allocation process in the US prioritized health care workers (HCWs) and residents of long-term care facilities, who were the first to be offered COVID-19 vaccines. As COVID-19 vaccination expands, it is important to continue to assess uptake of vaccination among HCWs across different racial/ethnic groups and ensure equitable allocation of vaccines. HCWs face occupational and community exposure to COVID-19, and they are essential to the public health response to this pandemic. Although the risk of occupational infection is low with proper use of personal protective equipment, studies have found that Black and Hispanic or Latino HCWs have a higher burden of infection, associated with increased community exposure to SARS-CoV-2.^9,10^ In a large study of 10 275 HCWs,^9^ community exposure and living in areas with increased COVID-19 prevalence were associated with most infections among HCWs. The odds of being infected with COVID-19 were increased 2-fold among Black HCWs and HCWs with multiracial backgrounds compared with White HCWs. The differential risk for community acquisition of SARS-CoV-2 among HCWs from racial/ethnic minority groups likely stems from historical and systemic practices that have disadvantaged communities of color and led to residential segregation, which is associated with increased exposure to SARS-CoV-2.^11^ Thus, uptake of COVID-19 vaccines is particularly crucial for HCWs living in communities with increased disease burden.

As an extension of our previous work,^12^ we analyzed data from a large survey of HCWs at 2 hospitals in Philadelphia in the weeks prior to the launch of the COVID-19 vaccine for HCWs. The purpose of this study was to describe COVID-19 vaccine hesitancy, defined as intent to decline vaccination, being unsure about vaccination, or intent to delay vaccination, across different racial/ethnic groups of HCWs and assess factors associated with vaccine hesitancy.

## Methods

Institutional review boards at each hospital in this survey study reviewed the protocol and determined that the study was exempt from further review and informed consent because the identity of participants could not readily be ascertained directly or through identifiers linked to participants. This study is reported following the American Association for Public Opinion Research (AAPOR) reporting guideline.

### Study Design and Participants

This was a cross-sectional survey study among HCWs. Eligible HCWs were asked to complete a confidential survey to assess their intention to receive a COVID-19 vaccine. HCWs with and without direct patient contact were eligible for this study if they were employees of hospital A (a children’s hospital) or hospital B (an adult hospital) or contracted by a third party to work at either hospital. Employees received an invitation to complete the survey using their work email. Reminders were sent via email once a week over a 3-week period in November and December 2020. HCWs who participated in COVID-19 vaccine clinical trials and those who did not report their race/ethnicity were excluded from our total sample in this analysis by race/ethnicity.

### Survey

The survey focused on intention to receive a COVID-19 vaccine among HCWs and their family, when they planned to be vaccinated after being offered the vaccine (ie, immediately or 3 month, 6 months, or 12 months later), concerns about vaccine safety, level of vaccine efficacy desired, vaccination history and children’s vaccination history, and demographic characteristics. When assessing intention to receive the COVID-19 vaccine, the following assumptions about the vaccine were presented: that it would be at least 50% effective and available at no cost and that vaccination would be voluntary. Employment characteristics included hospital of employment, role in the hospital, and years of employment. We also assessed HCWs’ exposure risk for COVID-19 in the hospital and in the community (ie, household and family exposure), as well as their history of COVID-19 infection. The surveys were developed and distributed using the Research Electronic Data Capture (REDCap) software version 10.9.4 (Vanderbilt University), and the data were stored in a secure server.

### Outcome Variable

Vaccine hesitancy was the outcome. HCWs were classified as being hesitant if they reported that they did not plan to receive a vaccine or were unsure about receiving a COVID-19 vaccine or if they planned on delaying receipt of the vaccine for 3 months, 6 months, or 12 months. HCWs who reported that they were planning to receive the COVID-19 vaccine as soon as it was available to them were classified as not hesitant.

### Covariates

#### Demographic Characteristics

Demographic characteristics of HCWs included age, race/ethnicity, sex, education level, and residential area categorized as urban, suburban, or rural. Race/ethnicity was categorized as White, Black or African American, Asian, Hispanic or Latino, and other or mixed race/ethnicity. The other or mixed race/ethnicity category included American Indian or Alaska Native, Pacific Islander or Hawaiian, or mixed race/ethnicity categories.

#### Employment Characteristics

Employment characteristics included hospital of employment (ie, hospital A or B) and whether the role was clinical with direct patient contact (eg, roles as physicians, nurses, or physical therapists), nonclinical with direct patient contact (eg, roles in transport or food and cleaning services), or nonclinical with no patient contact (eg, roles as administrative and research staff). We also collected years of employment (ie, <1 year, 2-4 years, or ≥5 years).

#### Risk of COVID-19 Exposure

We assessed occupational risk of COVID-19 exposure by the area of work in the hospital. COVID-19 units, emergency departments (EDs), and intensive care units (ICUs) represented high-risk areas for COVID-19 exposure compared with other floors and service units, where the risk of exposure was considered moderate. Low-risk areas included administrative offices, the home (ie, working remotely), and research labs. We assessed community exposure by asking if individuals had family or household members with past COVID-19 infection. We also asked HCWs if they had a prior COVID-19 diagnosis.

#### Being Up to Date With Routine Vaccinations

We asked if HCWs or their children were up to date on routine vaccinations. We additionally asked HCWs if they had refused vaccination for themselves or their children in the past.

### Statistical Analysis

We completed descriptive summary statistics (ie, frequencies and percentages) of COVID-19 vaccine hesitancy and HCW demographic and employment characteristics stratified by race/ethnicity. Differences in other variables across different racial/ethnic groups were evaluated by χ^2^ tests with a significance level of *P* = .05. All tests were 2-sided. We reported the reasons for vaccine hesitancy among HCWs who were hesitant and reasons for vaccine acceptance among HCWs who were not hesitant. We then conducted a 3-step hierarchical multivariable logistic regression using the following approach: the first model included demographic characteristics; the second model included variables in model 1, HCW employment characteristics, and risk for COVID-19 exposure; and the third model included variables in model 2 and being up to date on routine vaccinations. This approach was used to evaluate whether HCW employment characteristics and COVID-19 exposure risk (in model 2) and being up to date on other routine vaccinations (in model 3) were associated with race/ethnicity and vaccine hesitancy, after controlling for co-variates in the models.

Data analysis was conducted using SAS statistical software version 9.4. (SAS Institute). Data were analyzed from February through March 2021.

## Results

Among 34 865 eligible HCWs, 12 034 individuals (34.5%) completed the survey. After excluding 9 individuals (<0.1%) who received the COVID-19 vaccine in clinical trials and 1163 individuals (9.7%) who did not provide their race/ethnicity, our sample included 10 871 HCWs. Among 10 866 of these HCWs with data on sex, 8362 (76.9%) were women, and among 10 833 HCWs with age data, 5923 individuals (54.5%) were younger than age 40 years. (Percentages for demographic and clinical characteristics are among the number of respondents for each type of question.) There were 8388 White individuals (77.2%), 882 Black individuals (8.1%), 845 Asian individuals (7.8%), and 449 individuals with other or mixed race/ethnicity (4.1%), and there were 307 Hispanic or Latino individuals (2.8%).

### COVID-19 Vaccine Hesitancy by Race/Ethnicity

Overall, 5440 individuals (50.0%) had vaccine hesitancy. Vaccine hesitancy was highest among Black HCWs (732 individuals [83.0%; 95% CI, 80.4%-85.4%]) and Hispanic or Latino HCWs (195 individuals [63.5%; 95% CI; 57.9%-68.9%]), followed by HCWs with other or mixed race/ethnicity (244 individuals [54.3%; 95% CI, 49.6%-59.0%]), Asian HCWs (398 individuals [47.1%; 95% CI, 43.7%-50.5%]), and White HCWs (3871 individuals [46.2%; 95% CI, 45.1%-47.2%]) (*P* < .001) (Table 1). Most HCWs with vaccine hesitancy (3719 individuals [68.4%]) were those who were not planning to receive or were unsure about receiving the vaccine; 1721 individuals (31.6%) planned to delay the vaccine. The number and percentage who reported not planning to receive or being unsure about receiving the vaccine by race/ethnicity were as follows: 620 Black individuals (70.3%), 140 Hispanic or Latino individuals (45.6%), 185 individuals with other or mixed race/ethnicity (41.2%), 2555 White individuals (30.5%), and 219 Asian individuals (25.9%).

**Table 1.** Demographic and Clinical Characteristics of HCWs by Race/Ethnicity

Characteristic	HCWs, No./total No. (%)						*P* value
	White (n = 8388)^a^	Black (n = 882)^a^	Asian (n = 845)^a^	Other race/ethnicity (n = 449)^a^	Hispanic or Latino (n = 307)^a^	Total (N = 10 871)^a^	
**HCW characteristic**							
Vaccine hesitancy^b^							
Yes	3871/8388 (46.1)	732/882 (83.0)	398/845 (47.1)	244/449 (54.3)	195/307 (63.5)	5440/10871 (50.0)	<.001
No	4517/8388 (53.9)	150/882 (17.0)	447/845 (52.9)	205/449 (45.7)	112/307 (36.5)	5431/10871 (49.9)	
Demographic characteristic							
Age, y							
<40	4549/8363 (54.4)	391/878 (44.5)	511/843 (60.6)	298/444 (67.1)	174/305 (57.1)	5923/10833 (54.7)	<.001
40-64	3428/8363 (41.0)	470/878 (53.5)	325/843 (38.6)	135/444 (30.4)	128/305 (42.0)	4486/10833 (41.4)	
≥65	386/8363 (4.6)	17/878 (1.9)	7/843 (0.8)	11/444 (2.5)	3/305 (1.0)	424/10833 (3.9)	
Sex							
Men	1836/8384 (21.9)	142/882 (16.1)	270/845 (32.0)	116/449 (25.8)	69/306 (22.6)	2433/10866 (22.4)	<.001
Women	6509/8384 (77.6)	737/882 (83.6)	572/845 (67.7)	311/449 (69.3)	233/306 (76.1)	8362/10866 (77.0)	
Other^c^	39/8384 (0.5)	3/882 (0.3)	3/845 (0.4)	22/449 (4.9)	4/306 (1.3)	71/10866 (0.7)	
Education							
<Bachelor’s degree	805/8387 (9.6)	395/882 (44.8)	30/845 (3.6)	70/449 (15.6)	76/307 (24.8)	1376/10870 (12.7)	<.001
Bachelor’s or master’s degree	5342/8387 (63.7)	397/882 (45.0)	381/845 (45.1)	245/449 (54.6)	146/307 (47.6)	6511/10870 (59.9)	
Postgraduate degree	2240/8387 (26.7)	90/882 (10.2)	434/845 (51.4)	134/449 (29.8)	85/307 (27.7)	2983/10870 (27.4)	
Residential area							
Urban	3138/8387 (37.4)	493/881 (56.0)	418/845 (49.5)	252/448 (56.3)	164/307 (53.4)	4465/10868 (41.1)	<.001
Suburban	5049/8387 (60.2)	372/881 (42.2)	417/845 (49.4)	186/448 (41.5)	134/307 (43.7)	6158/10868 (56.7)	
Rural	200/8387 (2.4)	16/881 (1.8)	10/845 (1.2)	10/448 (2.2)	9/307 (2.9)	245/10868 (2.3)	
**HCW employment characteristic**							
Hospital of Employment							
Hospital A	5173/8388 (61.7	607/882 (68.8)	439/845 (52.0)	281/449 (62.6)	209/307 (68.1)	6709/10871 (61.7)	<.001
Hospital B	3215/8388 (38.3)	275/882 (31.2)	406/845 (48.1)	168/449 (37.4)	98/307 (31.9)	4162/10871 (38.3)	
Role in the hospital							
Clinical, with patient contact	5057/7818 (64.7)	287/806 (35.6)	509/805 (63.2)	235/407 (57.7)	138/276 (50.0)	6226/10112 (61.6)	<.001
Nonclinical, with patient contact	696/7818 (8.9)	203/806 (25.2)	32/805 (4.0)	45/407 (11.1)	31/276 (11.2)	1007/10112 (10.0)	
Nonclinical, with no patient contact	2065/7818 (26.4)	316/806 (39.2)	264/805 (32.8)	127/407 (31.2)	107/276 (38.8)	2879/10112 (28.5)	
Years of employment at hospital, y							
<1	650/8386 (7.8)	75/882 (8.5)	106/845 (12.5)	71/447 (15.9)	48/306 (15.7)	950/10866 (8.7)	<.001
2-4	2583/8386 (30.8)	306/882 (34.7)	324/845 (38.3)	185/447 (41.4)	133/306 (43.5)	3531/10866 (32.5)	
>5	5153/8386 (61.5)	501/882 (56.8)	415/845 (49.1)	191/447 (42.7)	125/306 (40.9)	6385/10866 (58.8)	
**Risk for COVID-19 exposure**							
Area with increased COVID-19 risk^d^							
High	2315/7791 (29.7)	165/772 (21.4)	235/794 (29.6)	132/420 (31.4)	86/276 (31.2)	2933/10053 (29.2)	<.001
Moderate	3685/7791 (47.3)	308/772 (39.9)	364/794 (45.8)	183/420 (43.6)	107/276 (38.8)	4647/10053 (46.2)	
Low	1791/7791 (23.0)	299/772 (38.7)	195/794 (24.6)	105/420 (25.0)	83/276 (30.1)	2473/10053 (24.6)	
Family or household member with COVID-19	1279/8388 (15.2)	188/882 (21.3)	58/845 (6.9)	68/449 (15.1)	68/307 (22.2)	1661/10871 (15.3)	<.001
Diagnosed with COVID-19	284/8388 (3.4)	44/882 (5.0)	14/845 (1.7)	15/449 (3.3)	16/307 (5.2)	373/10871 (3.4)	.008
**Routine vaccination status**							
Up to date on routine vaccinations							
Some, none, or unsure	166/8388 (2.0)	58/882 (6.6)	31/845 (3.7)	15/449 (3.3)	16/307 (5.2)	286/10871 (2.6)	<.001
All or most	8222/8388 (98.0)	824/882 (93.4)	814/845 (96.3)	434/449 (96.7)	291/307 (94.8)	10585/10871 (97.4)	
Ever refused a vaccine	443/8388 (5.3)	148/882 (16.8)	28/845 (3.3)	50/449 (11.1)	27/307 (8.8)	696/10871 (6.4)	<.001
Ever refused a vaccine for their children	280/4759 (5.9)	100/595 (16.8)	11/446 (2.5)	32/228 (14.0)	19/171 (11.1)	442/6199 (7.1)	<.001

Abbreviation: HCWs, health care workers.

^a^ The totals vary depending on the question and number of respondents. Reported statistics for the following variables are based on available data, excluding missing observations for plan to have immediate family members vaccinated (1684 individuals [15.5%]), age (38 individuals [0.3%]), sex (5 individuals [0.05%]), education (1 individual [0.01%]), residential area (3 individuals [0.03%]), role in the hospital (759 individuals [6.9%]), years employment (5 individuals [0.05%]), and area with increased COVID-19 risk (818 individuals [7.5%]).

^b^ Vaccine hesitancy was defined as not planning to receive or being unsure about receiving a COVID-19 vaccine or planning on delaying vaccination for 3 to 12 months.

^c^ Other included those who reported being transgender or binary, preferred not to say, or checked Other.

^d^ Occupational risk of COVID-19 exposure was classified as high exposure for HCWs working in COVID-19 units, emergency departments, or intensive care units; moderate exposure for HCWs working in nondesignated COVID-19 floors or services; and low risk for those working in administrative offices, research labs, or from home (ie, remotely).

### HCWs Demographic and Employment Characteristics by Race/Ethnicity

Demographic and employment characteristics for HCWs by race/ethnicity are presented in Table 1. Across all racial/ethnic groups, HCWs were mostly younger than age 40 years of age, and most held a bachelor’s or master’s degree. Most White HCWs (5049 of 8387 individuals [60.2%]) lived in the suburbs. Similar proportions among all 845 Asian HCWs lived in urban and suburban neighborhoods (418 individuals [49.5%] and 417 individuals [49.4%], respectively), while most Black HCWs, Hispanic or Latino HCWS, and HCWs with other or mixed race/ethnicity lived in an urban area (*P* < .001). Most HCWs held clinical jobs with direct patient contact, except for Black HCWs, among whom the largest percentage held nonclinical jobs with no patient contact (316 of 806 individuals [39.2%]) followed by clinical jobs with patient contact (287 of 806 individuals [35.6%]) (*P* < .001). Most HCWs had been employed for 2 or more years.

### HCWs’ Risk for COVID-19 Exposure by Race/Ethnicity

Across all racial/ethnic groups, the highest proportion of HCWs worked in areas of moderate risk for COVID-19 exposure, ranging from 107 of 276 Hispanic or Latino individuals (38.8%) to 3685 of 7791 White individuals (47.3%) (*P* < .001) (Table 1). Black and Hispanic or Latino HCWs were more likely to have had a COVID-19 diagnosis, with 44 Black HCWs (5.0%) and 16 Hispanic or Latino HCWs (5.2%) who had COVID-19 compared with 284 White HCWs (3.4%), 15 HCWs with other or mixed race/ethnicity (3.3%), and 14 Asian HCWs (1.7%) (*P* = .008). Similarly, Black HCWs (188 individuals [21.3%]) and Hispanic or Latino HCWs (68 individuals [22.2%]) were more likely to have family or household members with a history of COVID-19 compared with White HCWs (1279 individuals [15.2%]), HCWs with other or mixed race/ethnicity (68 individuals [15.1%]) and Asian HCWs (58 individuals [6.9%]) (*P* < .001).

### Being Up to Date on Routine Vaccinations by Race/Ethnicity

We also asked HCWs if they were up to date with all or most routine vaccinations (Table 1), and responses ranged from 824 Black HCWs (93.4%) to 8222 White HCWs (98.0%) (*P* < .001). The proportion of HCWs who reported that they had ever refused a vaccine for themselves varied from 28 Asian HCWs (3.3%) to 148 Black HCWs (16.8%) (*P* < .001), and the proportion of HCWs who ever refused a vaccine for their children varied from 11 of 446 Asian HCWs (2.5%) to 100 of 595 Black HCWs (17.2%) (*P* < .001).

### Reasons for COVID-19 Vaccine Hesitancy Among HCWs Who Were Hesitant

In Table 2, we report the frequency of reasons for vaccine hesitancy (from most to least common) by race/ethnicity and among 5440 individuals who indicated they were hesitant to receive the vaccine. Concerns among the entire surveyed population expressing hesitancy included side effects (4737 individuals [87.1%]), the newness of the vaccine (4306 individuals [79.2%]), not knowing enough about the vaccine (4091 individuals [75.2%]), the chance that it may not work (1535 individuals [28.2%]), and concerns that they may become infected with COVID-19 by receiving the vaccine (1170 individuals [21.5%]). Other reasons associated with outcomes from the disease, such as not being concerned about getting seriously ill from COVID-19 (455 individuals [8.4%]) or belief that COVID-19 is not as serious as people say it is (96 individuals [1.8%]), were of less significant concern.

**Table 2.** Reasons for Vaccine Hesitancy by Race/Ethnicity Among 5440 HCWs Who Were Hesitant

Reason for not getting vaccinated^a^	HCWs, No. (%)					
	White (n = 3871)	Black (n = 732)	Asian (n = 398)	Other race/ethnicity (n = 244)^b^	Hispanic or Latino (n = 195)	Total
Concern about side effects	3344 (86.4)	650 (88.8)	356 (89.5)	218 (89.3)	169 (86.7)	4737 (87.1)
Vaccine is too new	3015 (77.9)	627 (85.7)	309 (77.6)	197 (80.7)	158 (81.0)	4306 (79.2)
Don’t know enough about the vaccine	2898 (74.9)	575 (78.6)	292 (73.4)	181 (74.2)	145 (74.4)	4091 (75.2)
It may not work	960 (24.8)	316 (43.2)	116 (29.2)	83 (34.0)	60 (30.8)	1535 (28.2)
Concern about getting infected with COVID-19 from the vaccine	649 (16.8)	292 (39.9)	101 (25.4)	73 (29.9)	55 (28.2)	1170 (21.5)
Not concerned about getting seriously ill from COVID-19	304 (7.9)	83 (11.3)	24 (6.0)	26 (10.7)	18 (9.2)	455 (8.4)
Other	312 (8.1)	51 (7.0)	29 (7.3)	29 (11.9)	11 (5.6)	432 (7.9)
I do not like vaccines	68 (1.8)	63 (8.6)	5 (1.3)	13 (5.3)	7 (3.6)	156 (2.9)
COVID-19 outbreak is not as serious as some people say it is	70 (1.8)	13 (1.8)	4 (1.0)	6 (2.5)	3 (1.5)	96 (1.8)
I do not like needles	43 (1.1)	19 (2.6)	4 (1.0)	7 (2.9)	4 (2.1)	77 (1.4)
I won’t have time to get vaccinated	7 (0.2)	4 (0.6)	2 (0.5)	3 (1.2)	1 (0.5)	17 (0.3)
None of the above	30 (0.8)	9 (1.2)	3 (0.8)	2 (0.8)	3 (1.5)	47 (0.9)

Abbreviation: HCWs, health care workers.

^a^ HCWs could select more than 1 response. Those who were hesitant are those who would not take the vaccine immediately. Reasons are listed only for HCWs with vaccine hesitancy.

^b^ Other and mixed race/ethnicity included American Indian or Alaska Native, Pacific Islander or Hawaiian, and mixed race/ethnicity.

### Reasons for COVID-19 Vaccine Acceptance Among HCWs Who Were Not Hesitant

Reasons for vaccination among 5431 HCWs who were not hesitant are listed in Table 3 from most to least common reason for vaccine acceptance across different racial/ethnic groups. These reasons include the desire to protect their family (5225 individuals [96.2%]), themselves (5123 individuals [94.3%]), or their community (4574 individuals [84.2%]); to return to normal life (3998 individuals [73.6%]); to avoid becoming ill (3957 individuals [72.9%]); and to feel safe around people (3617 individuals [66.6%]).

**Table 3.** Reasons for Vaccine Acceptability Among 5431 HCWs Who Planned to Receive the Vaccine

Reason for vaccine acceptability^a^	HCWs, No. (%)					
	White (n = 4517)	Asian (n = 447)	Other race/ethnicity (n = 205)^b^	Black (n = 150)	Hispanic or Latino (n = 112)	Total
Want to protect my family	4361 (96.6)	423 (94.6)	194 (94.6)	138 (92.0)	109 (97.3)	5225 (96.2)
Want to protect myself	4288 (94.9)	409 (91.5)	185 (90.2)	139 (92.7)	102 (91.1)	5123 (94.3)
Want to protect my community	3809 (84.3)	380 (85.0)	172 (83.9)	118 (78.7)	95 (84.8)	4574 (84.2)
Life won’t get back to normal until most people are vaccinated	3342 (74.0)	329 (73.6)	150 (73.2)	96 (64.0)	81 (72.3)	3998 (73.6)
Want to prevent getting seriously ill from COVID-19	3292 (72.9)	334 (74.7)	137 (66.8)	113 (75.3)	81 (72.3)	3957 (72.9)
Allow me to feel safe around other people	299 (66.4)	307 (68.7)	129 (62.9)	110 (73.3)	72 (64.3)	3617 (66.6)
I want to travel again	2051 (45.4)	226 (50.6)	101 (49.3)	66 (44.0)	56 (50.0)	2500 (46.0)
My employer recommends the vaccine	811 (18.0)	54 (12.1)	28 (13.7)	29 (19.3)	17 (15.2)	939 (17.3)
I have a chronic condition, so it is important I am vaccinated	654 (14.5)	53 (11.9)	20 (9.8)	44 (29.3)	16 (14.3)	787 (14.5)
Doctor recommends the vaccine	633 (14.0)	41 (9.2)	22 (10.7)	27 (18.0)	11 (9.8)	734 (13.5)

Abbreviation: HCWs, health care workers.

^a^ Reasons for vaccine acceptability were assessed among HCWs who reported that they were planning on receiving the vaccine as soon as it was available.

^b^ Other and mixed race/ethnicity included American Indian or Alaska Native, Pacific Islander or Hawaiian, and mixed race/ethnicity.

### Adjusted Associations Between Race/Ethnicity and COVID-19 Hesitancy

Model 1 shows the association between race/ethnicity and vaccine hesitancy adjusting for demographic characteristics, such as age, sex, education, and area of residence (Table 4). Black HCWs had almost 5-fold higher odds of being hesitant to get a COVID-19 vaccine compared with White HCWs; the adjusted odds ratio (aOR) compared with White HCWs was 4.98 (95% CI, 4.11-6.03) for Black HCWs, 2.10 (95% CI, 1.63-2.70) for Hispanic or Latino HCWs, 1.48 (95% CI, 1.21-1.82) for HCWs with other or mixed race/ethnicity, and 1.47 (95% CI, 1.26-1.71) for Asian HCWs (*P* < .001). When adjusting for employment characteristics and COVID-19 exposure risk in model 2 and being up to date with prior vaccines in model 3, the aORs for hesitancy were decreased among Black HCWs and remained stable for other HCWs. The aORs for Black HCWs were 4.87 (95% CI, 3.96-6.00; *P* < .001) in model 2 and 4.48 (95% CI, 3.62-5.53; *P* < .001) in model 3. The aORs for other covariates are presented in Table 4.

**Table 4.** Odds of COVID-19 Vaccine Hesitancy by Health Care Worker Characteristic

Characteristic	Model 1^a^		Model 2^b^		Model 3^c^	
	OR (95% CI)	*P* value	OR (95% CI)	*P* value	OR (95% CI)	*P* value
Race/ethnicity						
White	1 [Reference]	<.001	1 [Reference]	<.001	1 [Reference]	<.001
Black	4.98 (4.11-6.03)		4.87 (3.96-6.00)		4.48 (3.62-5.53)	
Hispanic or Latino	2.10 (1.63-2.70)		2.08 (1.58-2.74)		2.03 (1.53-2.68)	
Asian	1.47 (1.26-1.71)		1.53 (1.30-1.80)		1.56 (1.32-1.83)	
Mixed or other	1.48 (1.21-1.82)		1.54 (1.23-1.92)		1.44 (1.15-1.80)	
Sex						
Men	1 [Reference]	<.001	1 [Reference]	<.001	1 [Reference]	<.001
Women	2.39 (2.15-2.65)		2.38 (2.12-2.67)		2.27 (2.02-2.55)	
Other or prefer not to answer	2.59 (1.53-4.40)		2.50 (1.41-4.42)		2.34 (1.31-4.19)	
Age, y						
<40	1 [Reference]	<.001	1 [Reference]	<.001	[Reference]	<.001
40-64	0.74 (0.68-0.81)		0.73 (0.66-0.81)		0.69 (0.62-0.78)	
≥65	0.42 (0.33-0.53)		0.43 (0.33-0.57)		0.42 (0.32-0.56)	
Education level						
<Bachelor’s degree	1 [Reference]		1 [Reference]		1 [Reference]	
Bachelor’s or master’s degree	0.62 (0.54-0.71)	<.001	0.62 (0.53-0.73)	<.001	0.63 (0.53-0.74)	<.001
Postgraduate degree	0.28 (0.24-0.32)		0.27 (0.22-0.32)		0.28 (0.23-0.34)	
Area of residence						
Urban	1 [Reference]	<.001	1 [Reference]	<.001	1 [Reference]	<.001
Suburban	1.45 (1.32-1.58)		1.38 (1.25-1.52)		1.35 (1.22-1.50)	
Rural	2.08 (1.56-2.76)		2.27 (1.65-3.13)		2.14 (1.54-2.96)	
Employment length, y						
<1	NA	NA	1 [Reference]	.01	1 [Reference]	.001
1-4	NA		1.27 (1.07-1.50)		1.31 (1.11-1.56)	
≥5	NA		1.29 (1.09-1.53)		1.38 (1.16-1.65)	
Level of patient care						
Clinical with direct patient contact	NA	NA	1 [Reference]	.03	1 [Reference]	.03
Some patient interaction	NA		0.84 (0.71-1.00)		0.83 (0.70-0.99)	
Nonclinical with no patient contact	NA		0.85 (0.74-0.98)		0.86 (0.75-0.99)	
Occupation COVID-19 exposure risk						
High	NA	NA	1 [Reference]	.39	1 [Reference]	.74
Moderate	NA		1.01 (0.91-1.12)		1.01 (0.91-1.12)	
Low	NA		1.11 (0.95-1.31)		1.07 (0.90-1.26)	
Employment site						
Hospital A	NA	NA	1 [Reference]	.90	1 [Reference]	.71
Hospital B	NA		0.99 (0.90-1.09)		0.98 (0.89-1.08)	
COVID-19 diagnosis						
Yes	NA	NA	1 [Reference]	.10	1 [Reference]	.05
No or unsure	NA		0.80 (0.61-1.04)		0.77 (0.59-1.00)	
Family COVID-19 diagnosis						
Yes	NA	NA	1 [Reference]	.27	1 [Reference]	.41
No or unsure	NA		0.93 (0.81-1.06)		0.95 (0.83-1.08)	
Refused prior vaccination						
Yes	NA	NA	NA	NA	1 [Reference]	<.001
No or unsure	NA		NA		0.29 (0.23-0.37)	
Refused prior vaccination for child						
Yes	NA	NA	NA	NA	1 [Reference]	<.001
No or unsure	NA		NA		0.31 (0.23-0.43)	
No children	NA		NA		0.32 (0.23-0.44)	
Up to date on most or all vaccinations						
Yes	NA	NA	NA	NA	1 [Reference]	.045
No or unsure	NA		NA		1.39 (1.01-1.91)	

Abbreviations: NA, not applicable; OR, odds ratio.

^a^ Model 1 adjusted for demographic characteristics, including age, sex, education, and area of residence.

^b^ Model 2 included Model 1 and adjusted for employment and COVID-19 risk characteristics, including years of employment, role in the hospital, area of employment in the hospital, prior COVID-19 diagnosis, and family or household member with COVID-19.

^c^ Model 3 included Model 2 and adjusted for being up to date with routine vaccinations, ever refusing a vaccine, and ever refusing a vaccine for their children.

## Discussion

According to the World Health Organization (WHO), vaccine hesitancy is defined as “the reluctance or refusal to vaccinate despite the availability of vaccines.”^13^ Vaccine hesitancy continues to be a considerable threat to global health and is among the top health priorities to tackle for WHO and the US.^13^ This survey study found that vaccine hesitancy was high among HCWs and there were substantial differences in COVID-19 vaccine hesitancy by race/ethnicity among HCWs, a population with potentially more access to accurate information about vaccine science and limited barriers to vaccine access. We found that, compared with White HCWs, hesitancy was highest among Black and Hispanic or Latino HCWs, followed by HCWs of other or mixed race/ethnicity and Asian HCWs. In the logistic regression models, Black HCWs had the greatest increase in odds of vaccine hesitancy compared with White HCWs, followed by Hispanic or Latino HCWs. This increase in odds remained high across regression models after adjusting for demographic variables, employment characteristics, and COVID-19 exposure risk, and it decreased among Black HCWs when adjusting for status regarding being up to date with routine vaccinations. In addition to reasons for vaccine hesitancy reported here, it is possible that mistrust of the health care system owing to historical mistreatment in research and medical care, particularly in the Black community, may contribute to hesitancy.^14^

Our results are consistent with prior studies finding that hesitancy toward a COVID-19 vaccine is particularly high among Black and Hispanic or Latino HCWs. In one survey of 3479 HCWs across 5 hospitals conducted shortly before our survey, 81% of Black HCWs and 70% of Hispanic or Latino HCWs were not willing to take the COVID-19 vaccine, mostly because they wanted to wait for safety data before deciding on vaccination.^15^ Another survey of 5287 HCWs conducted at approximately the same time as our survey found that 69.2% of Black and 60.7% of American Indian or Alaska Native HCWs disagreed with or were unsure about getting a COVID-19 vaccine.^16^ Data on vaccine hesitancy for Asian HCWs have been mixed^15,17,18^; however, reasons for vaccine hesitancy are similar to what has been reported for HCWs of other racial/ethnic groups and center on safety concerns.^17^ Interestingly, adjusting for employment characteristics and exposure to COVID-19 was not associated with much change in the aORs in our study, potentially suggesting that being in a health care environment and having first-hand knowledge of the disease were not significant factors associated with overcoming hesitancy.

Uptake of COVID-19 vaccines, even among HCWs, seems to lag among racial/ethnic groups in which vaccine hesitancy is high.^19^ A multisite cohort surveillance study^18^ conducted in January 2021, including HCWs from 20 EDs, found that 65.4% of Black HCWs received the COVID-19 vaccine compared with 88.5% of White HCWs. A March 2021 national survey from the Kaiser Family Foundation showed that of 1300 HCWs, 48% had not received the COVID-19 vaccine.^20^ The breakdown by race/ethnicity for individuals who received the vaccine was 39% for Black HCWs, 44% for Hispanic HCWs, and 57% for White HCWs. Access to COVID-19 vaccines was not an issue, given that 93% of hospital HCWs reported receiving the COVID-19 vaccine from their employers (vs at other vaccination sites) and 91% reported that it was very easy or somewhat easy to get vaccinated.

Strategies to increase COVID-19 vaccination among HCWs are needed. Although the Centers for Disease Control and Prevention and Food and Drug Administration have generally advised against COVID-19 vaccine mandates for vaccines authorized under the Emergency Use Authorization,^21^ some health care systems have mandated COVID-19 vaccination for their HCWs, using the influenza vaccine as a precedent.^22^ This approach may help accelerate vaccination for individuals who planned to delay or were contemplating vaccination; however, it will be important to monitor unintended outcomes associated with these mandates given that this approach may be associated with increased health and economic disparities if HCWs lose employment for refusing the vaccine.^23^ Other strategies, such as positive and culturally responsive messaging on COVID-19 vaccines and using vaccinated HCWs as vaccine ambassadors, should also be tested.

### Strengths and Limitations

Our study has several strengths. We conducted a large survey of HCWs before vaccine implementation in the US, and our findings highlight existing differences in COVID-19 vaccine hesitancy by race/ethnicity. Our sample represented HCWs from a broad range of backgrounds and roles. The 3-step hierarchal model we used allowed us to understand the relative associations of demographic characteristics, employment variables, COVID-19 risk exposure, and prior receipt of vaccines with COVID-19 vaccine hesitancy.

Our findings should be interpreted in the context of several important limitations. With a response rate of approximately 35%, there is a risk of selection bias and limited generalizability of the results. Although our sample was large, we surveyed respondents in 2 hospitals at 1 university-affiliated academic institution. Furthermore, because we used a convenience sample, we were not able to compare demographic characteristics of respondents and nonrespondents. It is possible that survey respondents were more likely to be hesitant and wanted their voices known. Our study measured intention to vaccinate prior to vaccine availability. As more people have gotten vaccinated and the acceptability of COVID-19 vaccines has increased in the general public,^24^ HCWs’ attitudes toward vaccination may have changed. Additionally, our survey did not include specific questions on attitudes, beliefs, or mistrust concerning the vaccine.

## Conclusions

This study found that approximately half of all HCWs were hesitant about COVID-19 vaccines, with the highest rates among Black and Hispanic or Latino HCWs. These results suggest that more work is needed to ensure confidence in COVID-19 vaccination, particularly among Black and Hispanic or Latino individuals, who are disproportionately impacted by the pandemic. Developing messaging emphasizing the individual, family, and community benefits of getting the vaccine and providing continued transparency on the safety profile of COVID-19 vaccines are simple approaches that may be rapidly disseminated across health care systems to improve vaccine acceptance among HCWs.

## References

[1] Holshue ML, DeBolt C, Lindquist S, ; Washington State 2019-nCoV Case Investigation Team. First case of 2019 novel coronavirus in the United States. N Engl J Med. 2020;382(10):929-936. doi:10.1056/NEJMoa2001191 32004427 PMC7092802

[2] Centers for Disease Control and Prevention. CDC COVID-19 data tracker. Accessed March 1, 2021. https://www.cdc.gov/coronavirus/2019-ncov/cases-updates/cases-in-us.html

[3] Centers for Disease Control and Prevention. Risk for COVID-19 infection, hospitalization, and death by race/ethnicity. Updated June 17, 2021. Accessed January 22, 2021. https://www.cdc.gov/coronavirus/2019-ncov/covid-data/investigations-discovery/hospitalization-death-by-race-ethnicity.html

[4] Oliver SE, Gargano JW, Marin M, . The Advisory Committee on Immunization Practices’ interim recommendation for use of Pfizer-BioNTech COVID-19 vaccine—United States, December 2020. MMWR Morb Mortal Wkly Rep. 2020;69(50):1922-1924. doi:10.15585/mmwr.mm6950e2 33332292 PMC7745957

[5] Oliver SE, Gargano JW, Marin M, . The Advisory Committee on Immunization Practices’ interim recommendation for use of Moderna COVID-19 vaccine—United States, December 2020. MMWR. Morb Mortality Wkly Rep. 2020;69(5152):1653-1656. doi:10.15585/mmwr.mm695152e1 33382675 PMC9191904

[6] Oliver SE, Gargano JW, Scobie H, . The Advisory Committee on Immunization Practices’ interim recommendation for use of Janssen COVID-19 Vaccine—United States, February 2021. MMWR Morb Mortal Wkly Rep. 2021;70(9):329-332. doi:10.15585/mmwr.mm7009e4 33661860 PMC7948932

[7] Reiter PL, Pennell ML, Katz ML. Acceptability of a COVID-19 vaccine among adults in the United States: how many people would get vaccinated? Vaccine. 2020;38(42):6500-6507. doi:10.1016/j.vaccine.2020.08.043 32863069 PMC7440153

[8] Callaghan T, Moghtaderi A, Lueck JA, Correlates and disparities of COVID-19 vaccine hesitancy. SSRN. 2020. doi:10.2139/ssrn.3667971

[9] Baker JM, Nelson KN, Overton E, Lopman BA, Lash TL, Photakis M, Quantification of occupational and community risk factors for SARS-CoV-2 seropositivity among healthcare workers in a large US healthcare system. medRxiv. Preprint posted online November 3, 2020. 33513035 10.7326/M20-7145 PMC7877798

[10] Wilkins JT, Gray EL, Wallia A, . Seroprevalence and correlates of SARS-CoV-2 antibodies in health care workers in Chicago. Open Forum Infect Dis. 2020;8(1):a582. doi:10.1093/ofid/ofaa582 33447642 PMC7787182

[11] Webb Hooper M, Nápoles AM, Pérez-Stable EJ. COVID-19 and racial/ethnic disparities. JAMA. 2020;323(24):2466-2467. doi:10.1001/jama.2020.8598 32391864 PMC9310097

[12] Kuter BJ, Browne S, Momplaisir FM, . Perspectives on the receipt of a COVID-19 vaccine: a survey of employees in two large hospitals in Philadelphia. Vaccine. 2021;39(12):1693-1700. doi:10.1016/j.vaccine.2021.02.029 33632563 PMC7885691

[13] Akbar R. Ten threats to global health in 2019. World Health Organization. Accessed March 1, 2021. https://www.who.int/news-room/spotlight/ten-threats-to-global-health-in-2019

[14] Egede LE, Walker RJ. Structural racism, social risk factors, and Covid-19—a dangerous convergence for Black Americans. N Engl J Med. 2020;383(12):e77. doi:10.1056/NEJMp2023616 32706952 PMC7747672

[15] Shekhar R, Sheikh AB, Upadhyay S, . COVID-19 vaccine acceptance among health care workers in the United States. Vaccines (Basel). 2021;9(2):119. doi:10.3390/vaccines9020119 33546165 PMC7913135

[16] Shaw J, Stewart T, Anderson KB, . Assessment of U.S. health care personnel (HCP) attitudes towards COVID-19 vaccination in a large university health care system. Clin Infect Dis. 2021;ciab054. doi:10.1093/cid/ciab054 33491049 PMC7929026

[17] Gadoth A, Halbrook M, Martin-Blais R, . Cross-sectional assessment of COVID-19 vaccine acceptance among health care workers in Los Angeles. Ann Intern Med. 2021;174(6):882-885. doi:10.7326/M20-7580 33556267 PMC7926184

[18] Schrading WA, Trent SA, Paxton JH, ; Project COVERED Emergency Department Network. Vaccination rates and acceptance of SARS-CoV-2 vaccination among U.S. emergency department health care personnel. Acad Emerg Med. 2021;28(4):455-458. doi:10.1111/acem.14236 33608937 PMC8013804

[19] Painter EM, Ussery EN, Patel A, . Demographic characteristics of persons vaccinated during the first month of the COVID-19 vaccination program—United States, December 14, 2020-January 14, 2021. MMWR Morb Mortal Wkly Rep. 2021;70(5):174-177. doi:10.15585/mmwr.mm7005e1 33539333 PMC7861480

[20] Kirzinger A, Kearney A, Hamel L, Brodie M. KFF/The Washington Post frontline health care workers survey. Accessed June 3, 2021. https://www.kff.org/report-section/kff-washington-post-frontline-health-care-workers-survey-vaccine-intentions/

[21] Rothstein MA, Parmet WE, Reiss DR. Employer-mandated vaccination for COVID-19. Am J Public Health. 2021;111(6):1061-1064. doi:10.2105/AJPH.2020.306166 33539177 PMC8101589

[22] Penn Medicine. Penn Medicine to require all health system employees to receive COVID-19 vaccine. Accessed June 4, 2021. https://www.pennmedicine.org/news/news-releases/2021/may/penn-medicine-to-require-all-health-system-employees-to-receive-covid19-vaccine

[23] Momplaisir F, Haynes N, Nkwihoreze H, Nelson M, Werner RM, Jemmott J. Understanding drivers of COVID-19 vaccine hesitancy among Blacks. Clin Infect Dis. 2021;ciab102. doi:10.1093/cid/ciab102 33560346 PMC7929035

[24] Szilagyi PG, Thomas K, Shah MD, . National trends in the US public’s likelihood of getting a COVID-19 vaccine—April 1 to December 8, 2020. JAMA. 2020;325(4):396-398. doi:10.1001/jama.2020.26419 33372943 PMC7772743

